# Adult asthma prevalence and trend analysis by urban–rural status across sociodemographic characteristics—United States, 2012-20

**DOI:** 10.1016/j.jacig.2023.100085

**Published:** 2023-02-08

**Authors:** Xiaoting Qin, Cynthia A. Pate, Hatice S. Zahran

**Affiliations:** Asthma and Community Health Branch, Division of Environmental Health Science and Practice, National Center for Environmental Health, Centers for Disease Control and Prevention, Atlanta, Ga

**Keywords:** Asthma prevalence, trend, urban-rural status, sociodemographic characteristics, US adult

## Abstract

**Background:**

Asthma prevalence estimates among adults are limited for urban–rural classification across sociodemographic characteristics.

**Objectives:**

This study examined current asthma prevalence and annual trends by 6-level urban–rural categories across sociodemographic characteristics among US adults.

**Methods:**

Asthma prevalence for 2020 and annual trends for 2012-20 were estimated using Behavioral Risk Factor Surveillance System data. The 2013 National Center for Health Statistics urban and rural categories were used to define urban–rural status.

**Results:**

Current asthma prevalence was higher in medium (9.7%; prevalence ratio 1.103 [95% CI 1.037, 1.174]) and small (9.9%; 1.111 [1.031, 1.197]) metro than in large fringe metropolitan (8.6%), was higher in micropolitan (10.2%) than in both large fringe (8.6%; 1.115 [1.042, 1.194]) and large central metropolitan (8.8%; 1.080 [1.001, 1.066]) areas. Prevalence by sociodemographic characteristics varied between urban–rural scheme; the prevalence was significantly higher among adults aged 55-64 years in micropolitan (11.9%), women in small metro (12.8%), and other race non-Hispanic in noncore (most rural) (13.6%) areas, adults without a high school diploma in micropolitan areas (13.8%), household income <100% of federal poverty level in micropolitan areas (15.7%), and adults with insurance coverage in micropolitan areas (10.3%) compared to the corresponding populations in other urban–rural categories. During 2012-20, an increasing trend in prevalence was observed only in medium metro areas, with an annual percentage change of 0.81.

**Conclusions:**

Asthma prevalence differed by 6-level urban–rural categories. These findings might be helpful in establishing effective asthma control programs and targeting resource allocation at the local level.

Asthma is one of the most common chronic illnesses in the United States, where about 21.0 million adults (8.4% of the population) had current asthma in 2020.^1^ Although asthma affects persons from all segments of the population, epidemiologic information continues to indicate that asthma disproportionately affects certain populations more than others. National asthma surveillance summary data show that current asthma was higher among boys aged <18 years, women aged ≥18 years, non-Hispanic Black persons, non-Hispanic multiple-race persons, Puerto Rican persons, and persons with low family incomes.^2^

Rural populations in the United States have well-documented health disparities, including higher asthma prevalence.^2^, ^3^, ^4^, ^5^, ^6^, ^7^, ^8^, ^9^ Studies focused on urban and rural areas and on location-specific demographic and environmental characteristics might contribute to differences in observed disease prevalence and severity.^9^, ^10^, ^11^, ^12^, ^13^, ^14^, ^15^ Residents in rural areas might have worse health outcomes, might face health care access barriers, and may disproportionately experience economic difficulties.^16^ Rural residents tend to have fewer health care providers available, live farther from health care services, and have been further hampered by hospital closures.^10^

The 2013 National Center for Health Statistics Urban–Rural Classification Scheme for Counties assigns each county 1 of 6 categories, from most urban to most rural, based on metropolitan statistical area (MSA) (4 metropolitan and 2 nonmetropolitan).^17^ Previous studies have shown differences in asthma prevalence by these 6 National Center for Health Statistics urban–rural categories but have not focused on differences among sociodemographic factors that could contribute to disproportionate distribution of the asthma population. Guo et al^4^ reported that across these 6 urban–rural categories, current asthma prevalence differed significantly in 19 states among adults. Current asthma prevalence in medium metropolitan, small metropolitan, micropolitan, and noncore areas was higher than in large central metropolitan and large fringe metropolitan areas.

Current asthma prevalence estimates among adults by sociodemographic characteristics are available at national and state levels,^1^^,^^4^^,^^18^ but very little information is available on asthma prevalence by urban–rural categories across sociodemographic characteristics. The aims of this study were to examine the prevalence of asthma in urban–rural categories across sociodemographic characteristics and to assess annual trends in asthma prevalence by urban–rural categories during 2012-20. The distribution of asthma prevalence by urban–rural categories across sociodemographic characteristics might lead to a better understanding of the relative contributions of place of residence on current asthma prevalence. Moreover, the findings might help to achieve US Department of Health and Human Services Healthy People 2030 objectives to improve respiratory health and social determinants of health, including the place in which a person lives,^19^^,^^20^ to reduce the overall burden of disease and disparities.

## Methods

### Study population

We analyzed data from the 2012-20 Behavioral Risk Factor Surveillance System (BRFSS) to examine current asthma prevalence (in 2020) and trends (2012-20) by urban–rural categories among US adults. BRFSS is the nation’s premier system of health-related telephone surveys that collect state data about US residents aged ≥18 years regarding their health-related risk behaviors, chronic health conditions, and use of preventive services. BRFSS collects data in all 50 states as well as the District of Columbia and 3 US territories. Descriptions of the BRFSS survey design and sampling, data collection, and weights have already been provided.^21^ The median BRFSS response rate from 2012 through 2020 varied from 45.2% to 49.9%.

### Variables

The BRFSS includes 2 questions to identify persons with current asthma. Respondents are considered to have current asthma if they answer “yes” to both of following questions: (1) Has a doctor, nurse or other health professional ever told you that you had asthma? and (2) Do you still have asthma?^21^

Each county’s urban–rural category was determined using the National Center for Health Statistics’ 2013 urban–rural classification scheme for counties, which are based on state and county Federal Information Processing Standards codes.^17^^,^^22^ This classification scheme assigns each US county to 1 of 6 categories, from most urban to most rural, as follows: (1) large central metropolitan (counties in MSAs of ≥1 million population containing the principal city), (2) large fringe metropolitan (counties in MSAs of ≥1 million population not containing the principal city), (3) medium metropolitan (counties in MSAs of 250,000-999,999 population), (4) small metropolitan (counties in MSAs of <250,000 population), (5) micropolitan (urban cluster population of 10,000-49,999), and (6) noncore (nonmetropolitan areas that did not qualify as micropolitan, including those without an urban cluster population of at least 10,000). The county identified for each BRFSS respondent was obtained through a data-use agreement. One of 6 levels of urban–rural classification was identified for the county of residence for each respondent. Institutional review board review was not required because the study involved secondary analysis of existing deidentified data.

### Statistical analysis

Participants who responded “don’t know/not sure,” refused to answer, or had missing responses to the asthma questions or sociodemographic variables were excluded from the analysis (≤7%). The weighted prevalence of current asthma was estimated by urban–rural classification across all sociodemographic characteristics. Those characteristics included age (18-24, 25-34, 35-44, 45-64, and ≥65 years), sex (male, female), and race and ethnicity (non-Hispanic White, non-Hispanic Black, Hispanic, and non-Hispanic other races that includes Asian and American Indian/Alaska Native). Characteristics also included educational attainment (less than high school graduate, high school graduate, some college, and college graduate or above), insurance coverage (yes, no), and poverty status (ratio of family income to federal poverty level [FPL]: family income <100% of FPL [poor], 100% to <200% of FPL [near poor], ≥200% of FPL [nonpoor], and unknown). The US Census Bureau’s federal poverty threshold is based on 2020 family income and size.^23^

SAS v9.4 (SAS Institute, Cary, NC) and SAS-callable SUDAAN 11.1 (RTI International, Research Triangle Park, NC) were used to account for the complex survey design and to calculate weighted prevalence estimates with standard errors and 95% CIs. Survey weights were used to adjust for nonresponse and to produce estimates generalizable to a participating state’s population.^21^ The chi-square test of independence was used to test for a relationship between 2 categorical variables. A 2-sided *t* test was used to determine the significant difference between the prevalence of current asthma among 2 subgroups. Adjusted prevalence ratios (PRs) with 95% CIs were estimated by conducting logistic regression analyses while adjusting for sociodemographic characteristics: sex, age, race/ethnicity, educational attainment, insurance coverage, and FPL. To minimize the probability of a type I error (the chance of being wrong when rejecting the null hypothesis, α), and because of the large sample size and multiple pairwise comparisons, alpha (significance level) was set to .01. Results were considered statistically significant if *P* ≤ .01. Results for annual trend were considered significant if *P* < .05.

Trends in asthma prevalence by urban and rural categories across sociodemographic characteristics were assessed by Joinpoint Regression v5.1.^24^ This software calculates annual percentage change and determines if the trend or trends are statistically significant at .05. Observed values were plotted on graph as dots; lines were plotted using the modeled Joinpoint Regression results.

## Results

After excluding participants with missing sociodemographic variables, 394,831 adult respondents from the 2020 BRFSS data were included in this study. The mean age was 54.4 years (data not shown); 48.7% were male and 62.0% were White (Table I). The sociodemographic distributions across urban–rural counties differed significantly, except for sex (*P* < .01 for all; Table I). Specifically, adults who lived in large central metropolitan counties were more likely to be younger (aged 25-34, 35-44 years), Black, Hispanic, a college graduate, living with a household income <100% of the FPL, or without health insurance coverage. In contrast, adults who lived in noncore (most rural) counties were more likely to be older (aged ≥54 years), White, and have a high school education or less, and were less likely to live with a household income between 100% to 200% of FPL.

**Table I:** Sociodemographic distributions by urban and rural classification in US adults aged ≥18 years—United States, 2020

Characteristic	No.	Distribution, % (95% CI)							*P* value (Wald χ^2^ test)
		Overall	Large central metro	Large fringe metro	Medium metro	Small metro	Micropolitan	Noncore	
Total	394,831	100%	30.8 (30.4-31.1)	24.8 (24.5-25.1)	20.6 (20.3-20.8)	9 (8.9-9.2)	8.4 (8.3-8.6)	6.4 (6.3-6.5)	<.001∗
Sex									.880
Male	180,936	48.7 (48.4-49.1)	48.4 (47.5-49.2)	48.9 (48.2-49.6)	48.8 (48.1-49.5)	49.2 (48.2-50.1)	48.9 (48.1-49.7)	49 (48.1-50.0)	
Female	213,895	51.3 (50.9-51.6)	51.6 (50.8-52.5)	51.1 (50.4-51.8)	51.2 (50.5-51.9)	50.8 (49.9-51.8)	51.1 (50.3-51.9)	51 (50.0-51.9)	
Age	386,618								<.001∗
18-24 years	25,059	12.3 (12.0-12.6)	12.0 (11.4-12.6)	11.7 (11.2-12.3)	13.3 (12.7-13.9)	14.2 (13.5-15.0)	12.2 (11.6-12.8)	10 (9.2-10.7)	
25-34 years	43,222	17.7 (17.4-18.0)	20.6 (19.9-21.3)	16.1 (15.6-16.7)	17.1 (16.5-17.7)	17.3 (16.5-18.1)	15.4 (14.7-16.0)	15.3 (14.5-16.2)	
35-44 years	50,074	16.3 (16.0-16.6)	17.5 (16.8-18.2)	16.6 (16.1-17.1)	15.8 (15.3-16.3)	15.1 (14.4-15.8)	15.3 (14.7-15.9)	14 (13.3-14.7)	
45-54 years	56,261	15.1 (14.8-15.4)	15.2 (14.6-15.9)	16.3 (15.8-16.8)	14.6 (14.1-15.1)	13.7 (13.1-14.3)	14.2 (13.7-14.8)	14.5 (13.8-15.1)	
55-64 years	75,981	16.6 (16.3-16.9)	15.4 (14.8-16.1)	17.4 (16.9-17.9)	16.6 (16.1-17.1)	16 (15.4-16.7)	17.7 (17.1-18.3)	18.4 (17.7-19.0)	
65+ years	136,021	22.0 (21.8-22.3)	19.2 (18.6-19.9)	21.8 (21.3-22.3)	22.6 (22.1-23.1)	23.7 (23.0-24.4)	25.3 (24.6-25.9)	27.9 (27.1-28.6)	
Race and ethnicity	385,884								<.001∗
White, non-Hispanic	295,619	62.0 (61.6-62.3)	44.1 (43.3-45.0)	65.6 (64.9-66.4)	65.8 (65.1-66.6)	74.4 (73.4-75.4)	78.9 (78.2-79.6)	80.9 (80.1-81.7)	
Black, non-Hispanic	30,109	12 (11.8-12.3)	16.1 (15.5-16.8)	11.9 (11.4-12.4)	10.9 (10.4-11.3)	8.5 (8.0-9.0)	7.3 (6.8-7.8)	7.8 (7.3-8.3)	
Other, non-Hispanic	28,851	8.8 (8.6-9.1)	13.4 (12.7-14.1)	8.5 (8.1-9.0)	6.8 (6.5-7.2)	5.3 (4.9-5.8)	5.1 (4.8-5.5)	4.6 (4.2-4.9)	
Hispanic	31,305	17.2 (16.8-17.5)	26.3 (25.4-27.2)	13.9 (13.3-14.6)	16.5 (15.8-17.2)	11.7 (10.7-12.6)	8.7 (8.2-9.2)	6.7 (6.0-7.4)	
Educational attainment	392,960								<.001∗
Not a high school graduate	25,540	12.4 (12.1-12.7)	13.8 (13.0-14.5)	9.4 (8.8-9.9)	12.0 (11.4-12.6)	12.9 (12.0-13.7)	13.9 (13.2-14.6)	16.2 (15.3-17.0)	
High school graduate	105,180	27.8 (27.4-28.1)	23.7 (22.9-24.4)	26 (25.4-26.6)	29.1 (28.5-29.8)	31.5 (30.6-32.4)	34.2 (33.5-35.0)	36.1 (35.2-37.0)	
Some college	109,619	30.7 (30.4-31.1)	28.8 (28.0-29.6)	30.6 (30.0-31.3)	32.2 (31.6-32.9)	32.7 (31.8-33.5)	32.4 (31.6-33.1)	30.3 (29.4-31.2)	
College graduate	152,621	29.1 (28.8-29.4)	33.8 (33.0-34.5)	34.0 (33.4-34.6)	26.6 (26.1-27.2)	23.0 (22.4-23.6)	19.5 (19.0-20.0)	17.5 (16.9-18.1)	
Insurance coverage									<.001∗
Yes	359,423	87.6 (87.3-87.8)	86.3 (85.6-86.9)	89.4 (88.9-89.9)	87.6 (87.1-88.1)	87.6 (86.8-88.3)	87.6 (87.0-88.1)	86.3 (85.5-87.1)	
No	33,361	12.4 (12.2-12.7)	13.7 (13.1-14.4)	10.6 (10.1-11.1)	12.4 (11.9-12.9)	12.4 (11.7-13.2)	12.4 (11.9-13.0)	13.7 (12.9-14.5)	
Ratio of family income to FPL†	394,831								<.001∗
<100%	40,039	12.9 (12.7-13.2)	15 (14.3-15.7)	9.1 (8.7-9.6)	13.2 (12.6-13.7)	13.4 (12.7-14.2)	13.9 (13.3-14.5)	15.2 (14.5-16.0)	
100% to 200%	67,820	17.2 (16.9-17.5)	16.2 (15.6-16.9)	13.7 (13.2-14.2)	18.5 (17.9-19.1)	19.5 (18.8-20.2)	21.6 (20.9-22.3)	21.7 (20.9-22.4)	
>200%	206,083	48.6 (48.3-49.0)	47.6 (46.7-48.5)	54.6 (53.9-55.3)	48.1 (47.4-48.8)	47.1 (46.2-48.0)	43.5 (42.7-44.3)	41.5 (40.6-42.4)	
Unknown	80,889	21.2 (20.9-21.5)	21.2 (20.5-21.9)	22.6 (22.0-23.2)	20.2 (19.7-20.8)	20 (19.3-20.7)	20.9 (20.3-21.6)	21.6 (20.8-22.4)	

Source: Centers for Disease Control and Prevention, 2020 BRFSS. Sample size comprised BRFSS respondents.

∗ Statistically significant at *P* ≤ .01.

† FPL is defined by the US Department of Health and Human Services’ poverty guidelines.^23^

In 2020, current asthma prevalence was 9.2% among adults who reside in all 50 US states and the District of Columbia. Prevalence was higher in the micropolitan area (10.2%), followed by medium (9.7%) and small (9.9%) metropolitan areas, which were all significantly higher than the prevalence in large central metropolitan (8.8%), large fringe metropolitan (8.6%), and noncore (9.0%) areas (Tables II and III). After adjusting for sex, age, race and ethnicity, educational attainment, insurance coverage, and ratio of family income to federal poverty, having current asthma was more likely to be reported in medium metropolitan (adjusted PR [95% CI] 1.103 [1.037, 1.174]), small metropolitan (PR 1.111 [1.031, 1.197]), and micropolitan (PR 1.115 [1.042, 1.194]) areas than in large fringe metropolitan areas; and also was more likely to be reported in micropolitan areas than large central metropolitan areas (PR 1.080 [1.001, 1.066]). Pairwise comparison showed no prevalence difference between the 2 largest metro areas (central vs fringe; *P* = .451) and between 2 nonlarge metropolitan areas (medium vs small; *P* = .601), but it did show a significant difference between 2 nonmetropolitan areas (micropolitan higher than noncore, at *P* < .01). Prevalence among adults living in noncore counties (9.0%) did not differ from that of persons living in large central metropolitan (8.8%; *P* = .626) and in large fringe metropolitan (8.6%; *P* = .218) areas (Tables III and IV).

**Table II:** Adult current asthma prevalence and adjusted prevalence rate by 6-level urban–rural categories in US adults aged ≥18 years—United States, 2020

Category	No.	Prevalence, % (95% CI)	Adjusted PR, % (95% CI)†
Overall	37366	9.2 (9.0-9.4)	
Large central metro	5737	8.8 (8.4-9.3)	1.05 (0.98-1.13)
Large fringe metro	7040	8.6 (8.2-9.0)	Reference
Medium metro	7799	9.7 (9.3-10.1)	1.10 (1.04-1.17)∗
Small metro	5309	9.9 (9.3-10.5)	1.11 (1.03-1.20)∗
Micropolitan	6000	10.2 (9.7-10.7)	1.12 (1.04-1.19)∗
Noncore	5481	9.0 (8.5-9.6)	0.98 (0.91-1.05)

Source: Centers for Disease Control and Prevention, 2020 BRFSS. Current asthma refers to respondents with a “yes” answer to both questions: (1) Has a doctor, nurse, or other health professional ever told you that you had asthma? and (2) Do you still have asthma?

∗ Statistically significant at *P* ≤ .01.

† Adjusted for sociodemographic characteristics of sex, age, race and ethnicity, educational attainment, insurance coverage, and ratio of family income to federal poverty. Categories are listed in Table I.

**Table III:** Adult current asthma prevalence by 6-level urban–rural classification for sociodemographic characteristics—United States, 2020

Characteristic	No.	Prevalence, % (95% CI)							*P* value (Wald χ^2^ test)
		Overall	Large central metro	Large fringe metro	Medium metro	Small metro	Micropolitan	Noncore	
Total	37,366	9.2 (9.0-9.4)	8.8 (8.4-9.3)	8.6 (8.2-9.0)	9.7 (9.3-10.1)	9.9 (9.3-10.5)	10.2 (9.7-10.7)	9.0 (8.5-9.6)	<.001∗
Sex									
Male	11,959	6.5 (6.2-6.7)	6.1 (5.5-6.6)	6.3 (5.8-6.8)	6.8 (6.3-7.3)	7.0 (6.2-7.8)	7.4 (6.7-8.1)	6.4 (5.7-7.0)	.029
Female	25,407	11.8 (11.4-12.1)	11.4 (10.7-12.2)	10.8 (10.2-11.4)	12.6 (11.9-13.2)	12.8 (11.9-13.6)	12.8 (12.1-13.6)	11.6 (10.7-12.5)	<.001∗
Age									
18-24 years	2596	10.4 (9.7-11.2)	10.6 (8.8-12.4)	9.5 (8.1-10.8)	11.1 (9.6-12.5)	10.6 (9.0-12.3)	11.4 (9.4-13.4)	9.2 (7.0-11.4)	.434
25-34 years	4111	8.8 (8.3-9.3)	8 (7.0-9.0)	8.4 (7.5-9.4)	9.1 (8.2-10.0)	10.3 (8.3-12.2)	10.0 (8.4-11.5)	9.9 (7.7-12.2)	.129
35-44 years	4810	8.8 (8.3-9.3)	8.3 (7.2-9.3)	8.4 (7.5-9.3)	9.9 (8.8-11.0)	9.8 (8.4-11.2)	9.7 (8.5-11.0)	7.5 (6.3-8.7)	.020
45-54 years	6015	9.8 (9.3-10.3)	8.5 (7.4-9.5)	9.4 (8.4-10.5)	11.1 (9.9-12.4)	11.6 (9.8-13.3)	10.6 (9.5-11.7)	10.4 (9.1-11.6)	<.001∗
55-64 years	7827	9.9 (9.4-10.5)	9.9 (8.5-11.3)	8.8 (8.0-9.6)	10.6 (9.5-11.7)	9.9 (8.9-10.9)	11.9 (10.5-13.2)	9.9 (8.8-11.1)	<.001∗
65+ years	11,402	8.3 (7.9-8.7)	8.9 (7.8-10.1)	7.8 (7.2-8.5)	7.9 (7.3-8.5)	8.6 (7.6-9.6)	8.9 (8.1-9.6)	8.0 (7.3-8.7)	.202
Race and ethnicity	36,553								
White, non-Hispanic	27,418	9.4 (9.2-9.6)	9.1 (8.4-9.7)	8.9 (8.5-9.3)	10.0 (9.5-10.5)	9.9 (9.3-10.5)	9.9 (9.4-10.5)	8.9 (8.3-9.5)	<.001∗
Black, non-Hispanic	3465	11.4 (10.7-12.1)	12 (10.8-13.2)	10.4 (9.1-11.7)	11.0 (9.5-12.5)	11.3 (9.4-13.3)	13.3 (10.4-16.3)	10.2 (7.9-12.4)	.314
Other, non-Hispanic	2991	8.9 (7.1-8.9)	7.3 (5.6-9.1)	6.3 (5.0-7.6)	9.4 (7.9-10.9)	10.6 (8.6-12.7)	11.6 (9.7-13.5)	13.6 (10.8-16.3)	<.001∗
Hispanic	2679	7.6 (6.9-8.2)	7.4 (6.3-8.4)	7.2 (5.9-8.4)	8.0 (6.8-9.1)	9.1 (6.2-12.0)	8.8 (6.6-10.9)	5.2 (3.4-7.1)	.109
Educational attainment	37,236								
Not a high school graduate	3052	9.9 (9.2-10.6)	8.6 (7.2-10.1)	9.5 (8.0-11.0)	9.9 (8.7-11.1)	11.4 (9.2-13.5)	13.8 (11.7-15.8)	10.2 (8.6-11.9)	<.001∗
High school graduate	9758	8.7 (8.3-9.0)	8.5 (7.5-9.5)	8.3 (7.6-9.0)	9 (8.2-9.7)	9.4 (8.5-10.4)	8.9 (8.1-9.7)	8.3 (7.6-9.1)	.348
Some college	11,519	10.6 (10.2-11.1)	10.9 (9.8-12.0)	9.9 (9.1-10.7)	11.0 (10.1-11.9)	10.8 (9.6-12.0)	11.0 (10.1-12.0)	10 (8.7-11.2)	.318
College graduate	12,907	7.9 (7.6-8.2)	7.5 (7.0-8.1)	7.5 (7.0-7.9)	8.9 (8.4-9.5)	8.6 (7.9-9.3)	8.6 (7.8-9.4)	7.6 (6.9-8.4)	<.001∗
Insurance coverage	37,193								
Yes	34,699	9.6 (9.4-9.8)	9.5 (8.9-10.0)	8.0 (8.5-9.3)	10.1 (9.6-10.5)	10.3 (9.6-10.9)	10.3 (9.8-10.8)	9.4 (8.8-10.0)	<.001∗
No	2494	6.5 (6.0-7.0)	5.1 (4.3-5.9)	6.1 (4.9-7.3)	7.5 (6.4-8.6)	7.7 (6.3-9.2)	9.6 (7.8-11.5)	6.5 (5.2-7.8)	<.001∗
Ratio of family income to FPL†	37,366								
<100%	6019	12.4 (11.7-13.1)	10.3 (9.0-11.6)	11.9 (10.4-13.5)	13.5 (12.2-14.9)	14.2 (12.3-16.1)	15.7 (14.1-17.3)	14.1 (11.7-16.4)	<.001∗
100% to 200%	7443	10.2 (9.7-10.7)	9 (8.0-10.1)	10.4 (9.3-11.6)	10.7 (9.8-11.7)	11.8 (10.3-13.4)	11.2 (10.1-12.3)	9.3 (8.3-10.3)	<.001∗
>200%	16,723	8.0 (7.7-8.3)	8.1 (7.5-8.8)	7.7 (7.3-8.2)	8.6 (8.0-9.2)	8.1 (7.3-9.0)	7.9 (7.2-8.6)	6.6 (6.1-7.2)	<.001∗
Unknown	7181	9.1 (8.6-9.5)	9.2 (8.0-10.5)	8.2 (7.5-9.0)	8.9 (8.1-9.8)	9.4 (8.3-10.4)	10.3 (9.1-11.5)	9.8 (8.6-10.9)	.064

Source: Centers for Disease Control and Prevention, 2020 BRFSS. Current asthma refers to respondents with a “yes” answer to both questions: (1) Has a doctor, nurse, or other health professional ever told you that you had asthma? and (2) Do you still have asthma?

∗ Statistically significant at *P* ≤ .01.

† FPL is defined by the US Department of Health and Human Services’ poverty guidelines.^23^

**Table IV:** *P* value of *t* test for pairwise comparison of current asthma prevalence by 6-level urban rural areas across sociodemographic characteristics—United States, 2020

Characteristic	Large metro	Nonlarge metro	Nonmetropolitan	Micropolitan				Noncore			
	Central vs fringe	Medium vs small	Micropolitan vs noncore	Micropolitan vs central	Micropolitan vs fringe	Micropolitan vs medium	Micropolitan vs small	Noncore vs central	Noncore vs fringe	Noncore vs medium	Noncore vs small
Total	.451	.601	.002∗	.001∗	.001∗	.162	.482	.626	.218	.044	.028
Sex											
Male	.519	.645	.023	.003∗	.008∗	.127	.412	.473	.869	.318	.224
Female	.197	.713	.034	.010∗	.001∗	.571	.882	.816	.155	.082	.061
Age											
18-24 years	.326	.701	.145	.582	.124	.813	.580	.320	.810	.157	.295
25-34 years	.557	.296	.976	.036	.093	.332	.822	.127	.228	.496	.829
35-44 years	.839	.944	.014	.084	.103	.884	.950	.352	.244	.005∗	.017
45-54 years	.188	.698	.842	.006∗	.150	.489	.340	.019	.252	.398	.282
55-64 years	.187	.358	.032	.050	.001∗	.164	.024	.986	.119	.383	.990
65+ years	.107	.251	.115	.921	.045	.064	.707	.188	.732	.888	.331
Race/ethnicity											
White, non-Hispanic	.638	.761	.010∗	.035	.002∗	.882	.874	.744	.929	.007∗	.026
Black, non-Hispanic	.089	.782	.087	.399	.073	.161	.263	.154	.834	.536	.427
Other, non-Hispanic	.342	.349	.243	.001∗	.001∗	.081	.508	.001∗	.001∗	.009∗	.093
Hispanic	.831	.481	.014	.248	.213	.531	.848	.046	.084	.013	.028
Educational attainment											
Not a high school graduate	.415	.250	.009∗	.001∗	.001∗	.002∗	.112	.155	.516	.750	.420
High school graduate	.779	.465	.284	.484	.250	.893	.403	.829	.940	.218	.071
Some college	.173	.726	.172	.834	.074	.996	.725	.278	.979	.168	.359
College graduate	.838	.507	.078	.030	.012	.473	.935	.823	.677	.006∗	.055
Insurance coverage											
Yes	.083	.587	.031	.034	.001∗	.528	.988	.876	.149	.085	.050
No	.179	.777	.006∗	.001∗	.002∗	.050	.117	.083	.695	.241	.202
Ratio of family income to FPL†											
<100%	.117	.573	.256	.001∗	.001∗	.040	.231	.007∗	.142	.705	.927
100% to 200%	.079	.222	.015	.008∗	.365	.538	.495	.729	.136	.047	.007∗
>200%	.338	.332	.006∗	.677	.659	.122	.680	.001∗	.004∗	.001∗	.003∗
Unknown	.174	.533	.501	.211	.004∗	.065	.2416	.546	.033	.2663	.632

Source: Centers for Disease Control and Prevention, 2020 BRFSS. Current asthma refers to respondents with a “yes” answer to both questions: (1) Has a doctor, nurse, or other health professional ever told you that you had asthma? and (2) Do you still have asthma?

∗ Statistically significant at *P* ≤ .01.

† FPL is defined by the US Department of Health and Human Services’ poverty guidelines.^23^

In comparing asthma prevalence by sociodemographic characteristics, the prevalence overall was significantly higher among women (11.8%) than men (6.5%); among Blacks (11.4%) than Whites (9.4%), Hispanics (7.6%), and other races (8.9%); among adults with health insurance (9.6%) than adults without health insurance (6.5%); among adults with a household income <100% FPL (12.4%) (poor) than those with an FPL of 100-200% (near poor) (10.2%) and those with an FPL of >200% (not poor) (8.0%) (*P* < .01 for all; Table III).

Further stratified analyses by urban–rural categories across the sociodemographic characteristics showed that current asthma prevalence was higher among men living in micropolitan areas (7.4%) than among men living in large central (6.1%) and large fringe (6.3%) metropolitan areas. The prevalence among women living in micropolitan (12.8%), small metro (12.8%), or medium metro (12.6%) areas was higher than among women living in large central (11.4%) and large fringe (10.8%) metropolitan areas (*P* < .01 for all; Tables III and IV).

Asthma prevalence did not differ significantly by urban–rural categories for adults aged 18-24, 25-34, or 35-44 years and adults aged 65 years or older. It did differ among adults aged 45-54 and 55-64 years. Prevalence among adults aged 45-64 years living in small metro (11.6%), medium metro (11.1%), and micropolitan (10.6%) areas were significantly higher than among adults in large central metro areas (8.5%), but no difference from fringe metro (9.4%) and in noncore (10.4%) areas. Asthma prevalence among adults aged 55-64 years living in micropolitan areas (11.9%) was significantly higher than among adults living in large fringe metro (8.8%), large central metro (9.9%), small metro (9.9%), and noncore (9.9%) areas (*P* < .01 for all; Tables III and IV).

Among race and ethnicity groups across urban–rural categories, asthma prevalence did not differ by urban–rural categories for Black or Hispanic adults. For White adults, prevalence was higher among those living in medium metro (10.0%), small metro (9.9%), and micropolitan (9.9%) areas than among those living in large fringe metro (8.9%) and noncore (8.9%) areas. For other-race adults, prevalence was significantly higher in noncore (13.6%) and micropolitan (11.6%) areas than in large central (7.3%) and large fringe metro (6.3%) areas (*P* < .01 for all; Tables III and IV).

Asthma prevalence did not differ by urban–rural categories for adults who were high school graduates or with some college education. Among adults without a high school diploma, prevalence was higher among those living in micropolitan areas (13.8%) than those living in large central metro (8.6%), large fringe metro (9.5%), and medium metro (9.9%) areas. Among adults with a college diploma, prevalence was higher among those living in medium metro areas (8.9%) than among adults living in noncore areas (7.6%).

For persons with or without health insurance coverage, asthma prevalence was higher in micropolitan (10.3% with insurance, 9.6% without insurance) and small metro (10.3% with insurance, 7.7% without insurance) areas than in large fringe metro areas (8.0% with insurance, 6.1% without insurance) (*P* < .01 for all; Tables III and IV).

Asthma prevalence among adults with household income <100% of FPL and living in micropolitan (15.7%) or in noncore (14.1%) areas was significantly higher than among those in large central metro areas (10.3%). In contrast, adults with a household income >200% of FPL and living in noncore countries had the lowest asthma prevalence (6.6%), which was significantly lower than the prevalence in the other 5 urban–rural level categories (*P* < .01 for all; Tables III and IV).

**Fig 1:**
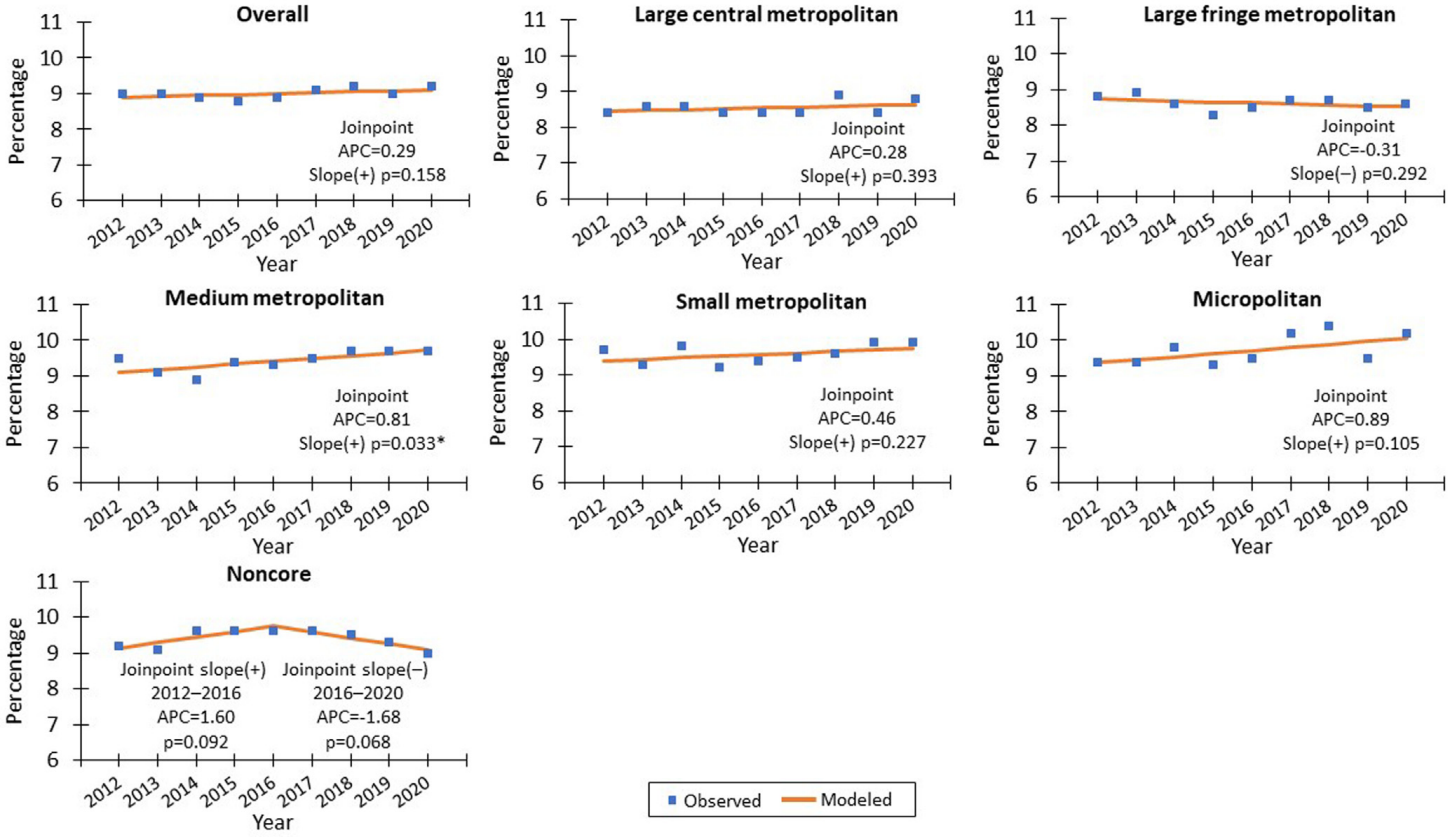
Prevalence of current asthma by urban–rural classification—United States, 2012-20. *APC,* Annual percentage change. ∗*P* value statistically significant at .05 level. Data source: National Center for Health Statistics, National Health Interview Survey, 2012-20.

Overall asthma prevalence did not change significantly from 2012 through 2020 (*P* = .158) (Fig 1). When we further explored the trends by urban and rural categories, we observed the significant increased trend only among adults living in medium metro areas, with an annual percentage change of 0.81 (slope *P* = .044).

## Discussion

This report presents results of a study that estimated current asthma prevalence and assessed prevalence trends by urban and rural categories for sex, age, race and ethnicity, educational attainment, insurance status, and family income ratio to FPL, revealing a difference in asthma prevalence according to urban–rural location. As indicated in our previous publications,^1^, ^2^, ^3^, ^4^^,^^18^^,^^25^ as well as in this study, current asthma prevalence differs by demographic factors, income, and geographic locations. Consistent with previous publications,^1^^,^^2^ not considering place of residence, asthma disproportionately affects certain populations more than others. Those disproportionally affected include women, Blacks, adults with health insurance, and adults living in a household with income <100% FPL. This study also determined that asthma prevalence differed by urban–rural status among these sociodemographic factors, suggesting that urban–rural geographic location plays an important role in the distribution of certain sociodemographic groups for asthma prevalence.

Trend analyses indicated that changes in asthma prevalence were mostly minor and nonsignificant during 2012-20 for all urban–rural areas. However, in medium metro areas, a small but significant increased trend was seen during 2012-20, with an annual percentage change of 0.81 (slope *P* = .04). Current asthma prevalence among US adults was higher among those living in medium metropolitan (9.7%), small metropolitan (9.9%), and micropolitan (10.2%) areas than among those living in large central metropolitan (8.8%) and large fringe metropolitan (8.6%) areas. A previous report also found that asthma prevalence was higher in medium and small metropolitan areas than in large central metropolitan areas.^2^

Asthma prevalence also was higher among adults living in micropolitan areas (10.2%) than in noncore areas (9.0%) at *P* < .01. The National Center for Health Statistics often uses the terms “noncore” and “rural” interchangeably; both micropolitan and noncore areas have also been known as “rural counties.” Noncore counties include nonmetropolitan areas that are not micropolitan and do not have a city, town, or urban cluster of 10,000 residents or more. Adults who lived in noncore counties were more likely to be older (aged ≥54 years), to be White, and to have a high school education or less, but less likely to live with a household income between 100% to 200% of FPL. We do not know with certainty which specific characteristics of urban–rural areas might be responsible for the differences in asthma prevalence between these 2 nonmetropolitan categories according to this study. The differences might partially be explained by differences in sociodemographic characteristics of the population,^22^^,^^25^ access to quality health care,^5^^,^^25^^,^^26^ and income.^25^, ^26^, ^27^

This study showed that asthma prevalence was higher in nonmetropolitan counties than in larger metropolitan counties among persons with the lowest ratio of household income to FPL, suggesting possible socioeconomic barriers to wellness. Asthma prevalence was also higher in micropolitan and small metro areas than in large fringe areas for those with and without health insurance. Residents of suburban areas (comparable to large fringe metropolitan) have been found to have higher-quality health care and better health outcomes.^28^ The finding of a higher percentage of adults who have asthma with health insurance coverage is consistent with prior reports that people with asthma are more likely to have health insurance than those without asthma.^29^

The findings of this study are subject to at least 3 limitations. First, the data were self-reported; therefore, the findings might be affected by social desirability and selective recall biases, possibly resulting in misclassification. However, BRFSS has been collecting data on a broad range of health topics, including asthma, since 1984,^21^ and a standardized asthma case definition for prevalence estimates has been used by most national and state surveys since 2001, including for BRFSS surveys. Second, for this report, we mainly used descriptive statistics, except for overall current asthma prevalence in urban–rural areas, considering that reporting estimates as observed in the population (without adjusting for covariates) is preferred for public health reporting and surveillance. Finally, cross-sectional survey data were analyzed, and associations are not necessarily causal. Variables other than those analyzed might contribute as well to certain observed differences

In conclusion, this study’s findings indicate that that current asthma prevalence was higher in medium metropolitan and small metropolitan areas than in large fringe areas, and was higher in micropolitan areas than both large central and large fringe metropolitan. The findings also show that the differences in current asthma prevalence among the sociodemographic characteristics varied across urban–rural categories. Moreover, this study identified socioeconomic and demographic disparities in asthma by considering place of residence. To our knowledge, ours is the first study in the United States to show that rural/suburban asthma prevalence is higher than that of large cities, adjusting for sociodemographic factors. These findings will help advance our understanding of asthma disparities between large metro, medium, and nonmetropolitan counties, leading to improved effectiveness in reducing the burden of asthma according to urban–rural classification. These location-specific findings will, we hope, provide valuable information to asthma control programs in developing targeted outreach programs and strategic resource allocation schemes to reduce the burden of asthma.
